# Persistent Effects of Musical Training on Mathematical Skills of Children With Developmental Dyscalculia

**DOI:** 10.3389/fpsyg.2019.02888

**Published:** 2020-01-10

**Authors:** Fabiana Silva Ribeiro, Flávia Heloísa Santos

**Affiliations:** ^1^Faculty of Education and Psychology (CEDH/HNL), Universidade Católica Portuguesa, Porto, Portugal; ^2^School of Psychology, University College Dublin, Dublin, Ireland

**Keywords:** musical training, developmental dyscalculia, numerical cognition, mathematical skills, melodic and rhythmic lessons

## Abstract

Musical training (MT) is perceived as a multi-sensory program that simultaneously integrates visual, aural, oral, and kinesthetic senses. Furthermore, MT stimulates cognitive functions in a ludic way instead of tapping straight into the traditional context of school learning, including mathematics. Nevertheless, the efficacy of MT over mathematics remains understudied, especially concerning longstanding effects. For this reason, this longitudinal study explored the impact of MT on numerical cognition and abstract visual reasoning using a double-blind and quasi-experimental design. We assessed two groups of children from primary schools, namely one with developmental dyscalculia [DD; *n* = 22] and another comprising typically developing children [TD; *n* = 22], who concomitantly underwent MT. Numerical cognition measurement was carried out at four different time points: Baseline (pre-MT assessment), mid-test (after 7 weeks of MT), post-test (after 14 weeks of MT), and follow-up (10 weeks after the end of MT). Significant interactions were found between time and group for numerical cognition performance, in which the DD group showed higher scores in number comprehension, number production at mid-test, and calculation at post-test compared to baseline. A key finding was that number production, number comprehension, and calculation effects were time-resistant for the DD group since changes remained on follow-up. Moreover, no significant differences over time were found for abstract visual reasoning for both groups. In conclusion, the findings of this study showed that MT appears to be a useful tool for compensatory remediation of DD.

## Introduction

Numerical cognition underlies daily-life activities and mathematical performance across our lifespan, and it has an impact on personal and professional development (Ancker and Kaufman, 2007). Numerical cognition includes six components; the *number sense*, an innate ability to recognize, compare, add, and subtract quantities without counting, the mental *number line*, which is an ordinal spatial representation of quantities and is built with experience (Dehaene, 1997), and the *numerical processing* and *calculation*, which are components developed during formal education. The numerical-processing is divided into *number comprehension*, responsible for understanding the nature of numeric symbols associated with their quantities, and *number production*, e.g., reading, writing, and counting numbers (McCloskey et al., 1985), and finally, the *calculation* component that is related to the performance of basic mathematical operations like addition, subtraction, multiplication, and division.

Deficits in *calculation* procedures, undeveloped problem-solving strategies, prolonged solution times, and higher inaccuracy rates are the core features of developmental dyscalculia (DD) (Geary, 1993; Skagerlund and Träff, 2016). In some cases, children with DD can also present symbolic processing deficits, e.g., difficulties with numerical digits and words or with their verbal and semantic representations (Kucian and Von Aster, 2015). Moreover, dysfunction in spatial reasoning is also present since quantities seem to be embodied in spatial formats, i.e., in the mental number line (Landerl, 2013). These deficits are associated with anomalies in brain functioning, for instance, some studies, comparing children with DD to typically developing control children, showed that the latter demonstrated higher activations in the intraparietal sulcus for number representations, while children with DD mostly activated medial frontal areas, revealing compensatory mechanisms (Kaufmann et al., 2011).

The prevalence of DD is around 3% and 7% in England and Israel, respectively (Butterworth, 2005; Shalev et al., 2005). However, it depends on the methodology and diagnosis criteria used (Devine et al., 2013). For this reason, prevalence rates may vary in different countries. For instance, in Brazil, Bastos et al. (2016) found a slightly higher prevalence (7.8% from a cohort of 2.893 children) compared to previously quoted studies. Nevertheless, Fortes et al. (2016) found 6% of prevalence in a cohort of 1.618 children from four different Brazilian states. Both Brazilian authors adopted cubes and vocabulary subtests of the Wechsler Intelligence Scale for Children (WISC-III) to assess the intellectual level. However, various mathematical screening protocols were used, for instance, Bastos et al. (2016) applied Grafman and Boller’s modified protocol, while Fortes et al. (2016) used the schooling achievement test (Stein, 1994). These divergences reveal the need to control the instruments selected, which should be standardized and not restricted to screening measures (Butterworth, 2005). Moreover, other factors should be considered, such as the socioeconomic classification of children’s families and schools (OECD, 2015), and the way children are assessed (individually or grouped).

Concerning DD prognosis, longitudinal studies have shown that DD is a persistent disorder that, when not appropriately assisted extends even beyond adolescence (Shalev et al., 2005; Mazzocco and Räsänen, 2013; Castro et al., 2014). Besides, it is essential to highlight that a longitudinal study performed by Ritchie and Bates (2013) was able to show that low socioeconomic and professional status in adulthood is not only a continuation of social status from one generation to the next but also a result of academic motivation and duration of education. For this reason, it is crucial to understand and create strategies that could improve or at least motivate these children to continue their academic studies.

Consequently, in recent years, approaches for remediation have been developed to enhance numerical cognition, for example, computer-assisted interventions (Kucian et al., 2011; Syah et al., 2015; Michels et al., 2018; Cheng et al., 2019), board games (Elofsson et al., 2016), and tutoring programs, i.e., the combination of conceptual features of numerical knowledge with counting skills (Iuculano et al., 2015), and even attentional training (Ashkenazi and Henik, 2012). These interventions produced improvements in the numerical cognition capacity, however, the effect was restricted to isolated numerical abilities such as number sense (Wilson et al., 2006), number line (Kucian et al., 2011; Käser et al., 2013; Elofsson et al., 2016), or number comprehension (Fuchs et al., 2013; Syah et al., 2015). Congruently, systematic reviews suggested that resistance to interventions may be an essential marker of DD (Monei and Pedro, 2017) and also that behavioral interventions have limited efficacy for children with DD (Kroeger et al., 2012). A factor, observed in those previous interventional-studies, was that most approaches embraced individual training, but it would be crucial to assess the effect of group settings since they would reinforce the social context and children’s well-being (Koelsch, 2010), which likewise might potentiate neuroplasticity (Davidson and McEwen, 2012).

### A Recent Cognitive Remediation Approach: Musical Training

Music is an enriching activity, and it seems to be an ideal tool to nourish human cognition (Koelsch and Siebel, 2005; Koelsch, 2010). Furthermore, music science is a field of research that investigates the impact of music on cognition. For instance, the “4E cognitive science” framework, has been recently adopted in music research by some authors (Schiavio and Altenmüller, 2015; Krueger, 2018; van der Schyff and Krueger, 2019). “4E” stands for Embodied, Embedded, Extended, and Enactive – four overlapping principles that help describe mental life in a novel and fascinating way. These principles challenge more traditional accounts of cognition (including music cognition) by emphasizing the fluid integration of neural, bodily, and environmental factors (Schiavio and van der Schyff, 2018; Ryan and Schiavio, 2019). For instance, researchers working from an *Embodied* perspective might offer novel tools to analyze their data, which more consistently integrate aspects of functions and processes distributed across the whole bodies of their participant(s) (Gallagher, 2005). In the case of *Embedded* cognition, environmental factors, such as cultural ones, are highlighted (Donald, 1991). With regard to *Extended* cognition, the authors argue that mental processes can be functionally coupled with tools and devices of their environment, generating novel opportunities to facilitate different cognitive tasks (Clark and Chalmers, 1998). Finally, the *Enactive* perspective describes cognition in terms of situated action, positing a deep continuity between mind and life (Varela et al., 1991; Thompson, 2007).

Musical training (MT) involves perception and action, which are mediated by a sensory, motor, and multimodal combination of diverse brain regions (Zatorre et al., 2007; Schlaug et al., 2010). Due to this multimodal characteristic, music intervention has the potential to change brain architecture that might be accompanied by cognitive changes. Although neuroanatomy (gross morphology of auditory cortex) has been shown to be extremely stable from childhood to adolescence in a longitudinal study (Seither-Preisler et al., 2014), other studies observed changes related to brain activation, as assessed by functional magnetic resonance imaging (Oechslin et al., 2013), electroencephalography (James et al., 2017), neural connectivity measured by diffusion tensor imaging (Moore et al., 2014), and cortical thickness (Hudziak et al., 2014).

Furthermore, music processing and mental calculations, such as addition and subtraction, seem to be connected to complex reasoning and to trigger similar brain pathways in both the prefrontal cortex and the parietal lobe (Schmithorst and Holland, 2004).

Few studies have been devoted to the study of the effects of MT on numerical cognition systems. However, some studies have proposed an association between music and executive functions, consequently influencing math skills (Dumont et al., 2017; Azaryahu et al., 2019; Guhn et al., 2019), and also the spatial-temporal reasoning (Rauscher et al., 1994; Graziano et al., 1999), which is essential to process number magnitude since it is spatially represented in the mind (Dehaene et al., 1993). In line with this, studies demonstrated that MT leads to improvements in spatial abilities in pre- and elementary school children boosting their learning of specific math concepts, such as counting, proportions, and fractions (Graziano et al., 1999; Silva et al., 2017; Arias-Rodriguez et al., 2019). If MT increases spatial-visual abstract reasoning, this practice might also improve comprehension about geometry, proportional reasoning, pattern recognition, ratio fractions, and subdivisions (Vaughn, 2000; Schlaug et al., 2005).

The associations between MT and improvement in mathematical skills is often explained by functional brain connectivity (Fingelkurts et al., 2005) and distributive brain processing (McIntosh, 2000). The first is related to the connectivity and organization of brain activity among diverse neuronal assemblies that share functional properties (Fingelkurts et al., 2005), the latter proposes that brain areas are structurally interrelated, and process information in a distributed way (McIntosh, 2000). Previous studies revealed that MT could enhance essential circuits for mental arithmetic such as left and right planum temporale related to temporal speech cue discrimination (Elmer et al., 2016), the left dorsolateral prefrontal cortex (Hudziak et al., 2014), and the intraparietal sulcus (Wan and Schlaug, 2010). Brain activations in these areas are consistent with the enhancement of numerical cognition and spatial reasoning (Gromko, 2004).

Regarding the results of behavioral studies that explored the effects of MT on numerical cognition, Silva et al. (2017), comparing two preschoolers gender-balanced groups, one that underwent eight MT sessions and another that did not receive the training, found that only the group that underwent the MT improved in counting, addition, and subtraction abilities. Moreover, Sanders (2012, 2018) found, in a quasi-experimental design with a MT involving clapping, tapping, and movement on a regular weekly basis during 9 to 10 months, improved mathematical performance such as counting and calculation in preschool and primary school children. She also showed that when a music- mathematics link was established, this effect slightly increased.

Nevertheless, the impact of MT has recently been challenged by specialists in the music psychology field (Schellenberg and Weiss, 2013; Sala and Gobet, 2017; Schellenberg, 2019). A meta-analysis performed by Sala and Gobet (2017) showed that the training effects in children with typical development (TD) and young adolescents’ cognitive or academic skills might not be reliable, and those previous positive findings were probably due to confounding variables.

Although, there are longitudinal studies, testing neurological and behavioral effects of MT on clinical samples of children with attention-deficit (hyperactivity) disorder (Seither-Preisler et al., 2014; Serrallach et al., 2016), and with dyslexia (Serrallach et al., 2016), very few studies have investigated the MT effects on learning disorder related to numerical abilities, such as DD (Esteki, 2013; Ribeiro and Santos, 2017; Arias-Rodriguez et al., 2019).

Moreover, MT may be an interesting option for remediation since it stimulates cognitive functions in a ludic way (Ribeiro and Santos, 2015), without tapping directly into the traditional context of math learning (Ribeiro, 2013; Ribeiro and Santos, 2017). In fact, it has been associated with near and far transfer in cognitive skills (Schellenberg, 2004; Miendlarzewska and Trost, 2013). In this context near transfer is related to beneficial effects in similar functions trained in MT, e.g., decode prosodic cues in speech (Thompson et al., 2004), while far transfer, for instance, can test the effects of MT in the IQ capacity (Schellenberg, 2004) or numerical cognition (Ribeiro and Santos, 2017).

With regard to a DD sample, Ribeiro and Santos (2017) carried out 14 sessions of MT for two groups of primary school children, one with low numerical abilities and one with typical-numerical skills. Results revealed that children with low achievement improved their numerical cognition performance, especially for number production capacity compared to normative data at post-MT, which consisted of singing, solfeggio, rhythmic, and melodic techniques (Ribeiro and Santos, 2012, 2017). In the present study, we aimed to extend the findings presented by Ribeiro and Santos (2017) by assessing children throughout the MT development and 10 months after the end of MT to evaluate whether the participants outcomes were stable over time. To the best of our knowledge, no other study has carried out a follow-up test regarding the persistence of MT effects on numerical cognition, especially in children with neurodevelopmental disorder.

We chose to apply an MT including singing, objects as percussion, and corporal movements due to the following reasons: (i) Firstly, because music is not part of the public school curriculum, and schools do not have available musical instruments or even a budget to buy them; (ii) Secondly, this study was developed in a countryside community of Brazil and families with low income neither have financial resources to purchase musical instruments nor traditionally acquire musical education. For these reasons, our MT seemed suitable in the context of developing countries since it is based on ecological resources, i.e., the MT sessions use activities and materials that are from children’s real-world resources (Shadish et al., 2002). These materials include recognition of sound in their environment, inside the classroom and in the surrounding areas (Ribeiro and Santos, 2017). In this context, music can be taught in an organic way, which means that children apply natural resources, such as their voices and bodies (for clapping and body games and movements) (Sanders, 2018).

In general, this study aimed to examine whether MT increases specific components of numerical cognition abilities in children with DD from a developing country. Specifically, we sought to assess the effect of a brief MT on DD children’s numerical cognition contrasting with children with TD. Initially, the design of the study comprised the two groups of DD and TD children (between-subjects factor), which received the same treatment over time (within-subjects factor). Moreover, we compared the DD group results to the normative data of Santos et al. (2012) by visual inspection to clarify whether they would have an equivalent performance at post-test, revealing improvement after MT.

We hypothesize that over the brief MT children with DD will improve performance that specifically concerns numerical cognition. In contrast, no substantial improvements are expected for the TD group. Furthermore, we anticipate that the TD group will score on numerical cognition tasks according to their age group when compared to the normative sample of Santos et al. (2012).

## Materials and Methods

### Study Design

**FIGURE 1:**
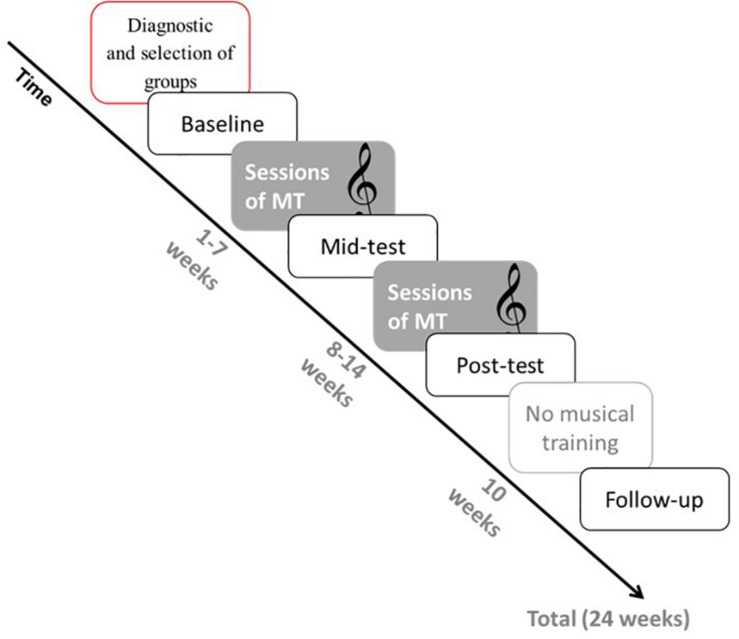
Schematic illustration of the experimental paradigm. MT, musical training.

This is longitudinal and double-blind research with a mixed design in which, firstly, we compared the groups’ baseline with the three-time points after the start of MT: mid-test in the middle of MT, post-test at the end of MT, and follow-up after 10 months. Secondly, the DD and TD groups were compared in the four different time points (Figure 1). All neurocognitive assessments were conducted by a psychologist blind to the intervention status of the children, and the MT was tutored by a music teacher who has had formal musical education and was specially invited to develop the MT. Moreover, he was unaware of the diagnosis of the children, and the children that participated in the MT were blind to their diagnosis.

### Participants

The participants were selected from a total cohort of 407 students enrolled in the 3rd school year of four different public schools in the countryside of São Paulo State, in Brazil. From the total cohort, we screened only those children indicated by the teacher as having mathematical difficulties confirmed by grades. For this reason, 223 children (112 boys, aged 8) underwent the screening phase, in which they were assessed in writing, arithmetic, and reading skills with the schooling achievement test (SAT; Stein, 1994). From 223 children, twenty-two out of 201 children fulfilled the criteria for DD and performed the MT (DD, 17 boys, *M*_age_ = 99.18, *SD*_age_ = 3.51 months). The DD was indicated by a cut-off value of <9 points in the arithmetic’s subtest of SAT (Stein, 1994), which means a substantial delay according to the criterion for the diagnosis of DD by the International Classification of Diseases (ICD-10, F81.2; World Health Organization [WHO], 2018). Their performance on an arithmetic subtest of SAT corresponded to one grade below TD group, which means a substantial delay according to the criterion for the diagnosis (ICD-10, F81.2; World Health Organization [WHO], 2018). Besides, an objective criterion for the DD diagnosis was assured by the cut-off of 1.5 SD below the mean age in the Zareki-R (Rotzer et al., 2009). In the present study, using the two-phase procedure, i.e., screening, and neuropsychological assessment (Shalev et al., 2005), the prevalence of DD was 5.4%, comprising more boys (60%) than girls (Bastos et al., 2016).

Therefore, for our TD, we selected a convenience sample of 29 from the 223 children screened previously, who had no deficit or delay in any subtest of the SAT or Zareki-R; however, three children dropped out the MT, and four were excluded after exploratory statistical analyses determined they were outliers (higher results in Zareki-R); then, data of twenty-two participants (18 boys) were included in the analyses. All participants were native Portuguese speakers and non-bilingual. None of these children presented emotional disorders, motor difficulties, speech and hearing impairments, or had a neurological and psychiatric diagnosis, based on the parent’s and the teacher’s reports. All children enrolled in the present study exhibited average visual abstract reasoning capacity assessed by Raven’s Colored Progressive Matrices Test according to normative criteria (percentile ranging from 40 to 74; Angelini et al., 1999), which was an inclusion criterion. For demographic characteristics, see Table 1.

**TABLE 1:** Means (and standard deviation) of demographic characteristics of DD and TD groups at baseline.

**Baseline**	**DD (*n* = 22)**	**TD (*n* = 22)**	***t***
SES	23.31 (4.88)	24.44 (6.40)	0.50
Age (years)	8.26 (0.29)	8.38 (0.30)	–1.28
CPM	69.27 (18.13)	74.55 (15.27)	–1.04
**Schooling achievement test**			
Writing	22.05 (2.15)	22.50 (2.76)	–0.61
Arithmetic	5.68 (1.49)	10.91 (1.45)	11.81^∗∗^
Reading	62.09 (2.54)	62.55 (3.14)	–0.53
Total	90.27 (4.58)	95.73 (6.33)	−3.27^∗^

*DD, children with developmental dyscalculia; TD, typical development children which underwent MT; *n*, number of participants; SES, socioeconomic status; CPM, Scale Raven’s colored progressive matrices. ^∗^*p* < 0.01.^∗∗^*p* < 0.001.*

Socioeconomic status was assessed by the Brazilian Association of Marketing Research Institutes Scale that stratifies the socioeconomic status in five classes, from A/richest to E/poorest (Associação Brasileira de Empresas de Pesquisa [ABEP], 2008). In the present study, mean socioeconomic status was classified as C/middle class, corresponding to 4 to 10 minimum monthly wages – 2,488 BRL to 6,220.00 BRL (∼ 1,141.28 USD to 2,853.21 USD). SES means, and standard deviations are displayed in Table 1.

The MT was conducted with five mixed (DD and TD) and balanced (age and gender) training classes once a week for 60 min (two classes with *n* = 8, two classes with *n* = 9, and one class with *n* = 10). The number of participants per class was small to enable active participation. The selection of the MT classes and the initial activities were assigned by pseudo-random criteria related to DD diagnosis and gender. The distribution took into account their classroom period to avoid interference in their regular classes, the training was performed in a different time of formal schooling classes. For example, a control child and a child with DD paired by sex and class time were allocated in the same MT group.

### Procedure

Four schools were chosen to be venues of this study according to the National Institute of Studies, and at the Brazilian Development Index of Primary Education that classifies the quality of education system performance from 0 to 10. The quality of the schools selected for this study was classified as ≥6 points, which meets the Organization for Economic Co-operation and Development standard (INEP, 2009), therefore, possible deficits should not be taken into account as pedagogical failures. Ethics committee approval was obtained by the UNESP, São Paulo State University (process number: 1367/2011). Furthermore, written consent was obtained from the participating schools and the parents/guardians of the children. We also asked for children’s assent to take part in the study.

During the screening phase, and to avoid disturbing the progress of classes, writing, arithmetic, and reading skills (SAT) were individually assessed in a quiet room at a schedule that did not coincide with that of regular classes. Then the selected children completed the Baseline (diagnosis phase), and after that, they were assigned to the MT. Group allocation is described in *Musical Training Paradigm*. The children participated in the MT for the first seven weeks, and, at this time, a mid-test was conducted. Immediately after completing fourteen weeks of training, a post-test was administered for the DD and the TD groups. A follow-up measure of all trained children was obtained 10 months after the end of the MT.

Children were assessed in their schools in a single, on average, 60-min session in a quiet room with pauses to avoid fatigue. The pencil-and-paper tasks were performed before the computerized ones. The baseline protocol of neurocognitive assessment was also used in the subsequent time points. Moreover, we also controlled the formal math classes with the participants’ teacher to make sure that the same math content was being taught during MT classes.

### Musical Training Paradigm

There were seven sessions for each set of activities, in which three classes (2 classes with *n* = 8, one class with *n* = 9) begun with the melodic activities, while the others (1 class with *n* = 9 and one class with *n* = 10) started with rhythmic activities. The mixed groups’ selection and the initial allocation onto rhythmic or melodic activities were random and balanced for each group. For all classes, the mid-test was carried out before the switch to the set of activities.

Our MT was grounded on active musical learning methodologies, such as Willems, Suzuki, Dalcroze Eurhythmics, and Suzuki methods, which combine music, movement, and speech into lessons to make it analogous to a setting where children play (Goulart, 2000). For instance, the activities selected from these musical methodologies are arranged in Table 2. A brief description of each session is presented below (Examples of a complete melodic and rhythmic sessions are given for the first lesson).

**TABLE 2:** Elements used from each musical education methodology in the musical training.

**Authors**				

	**Willems**	**Dalcroze**	**Orff**	**Suzuki**
**Parameters**				
Musical activities	Listening, singing, and body movement are developed by reference to the natural movement (march, run, and hop)	Listening, body movements, aim to enhance coordination among eyes, ears, mind, and body	Singing, talking, dancing, rhymes, clapping and making sounds with objects and the body	Imitation, global attention of the child, and their auditory sensitivities, visual, and kinesthetic
Rhythm	Rhythm is connected to the prosodic accent with the use of wooden sticks. Later the activity gradually takes on the characteristic of abstraction and consciousness	Each musical element: accentuation, phrasing, dynamics, pulse, tempo, and meter – can be studied by looking at a given movement	Progressive learning of rhythm elements as patterns, pulse, and time). Ostinato (rhythmic pattern, spoken, or sung)	Learning of changes and distinction of pitch and melodic structures
Melody	Melody is used in all the progression starting from the panchromatic sound movement all the way to the song (synthesis)	Aspects of melody are studied through the analysis of sounds emitted through body motion	The experience of the almost universal melodic contours is given by the pentatonic pitches (CDEGA). To interpret musical histories and improvisations	Used the identification and maintenance of repeated rhythmic pattern
Learning methods	Imitation, invention, and improvisation	Improvisation through movements suggested by students or/and the musical instructor	Improvisation by body percussion and musical rhythms sung. The instruments were not included in this study	Toning ability (student’s ability to produce and recognize a timbre and the tone of a sound). The use of an instrument was not included in this study

### Melodic Activities

1st Lesson: Introducing the concept of sound and silence, recognition of sounds in different environments. For this purpose, in the first lesson, the children were asked to describe all the sounds that they could identify inside and outside of the classroom. Thereafter, they were asked to reproduce the sounds described and later to detect and sing the intensity and tonal range in words said out loud in the classroom and through recorded audios of environmental sounds, such as horns, dog barks etc., as well as musical notes from instruments such as the piano and guitar.

2nd Lesson: Working with animal sounds of the city and forest, the contrast between treble, midrange and bass sounds, recognition of animal sounds, and their differentiation.

3rd Lesson: Recognizing various musical instruments, exemplifying the concept of timbre and its distinction, the use of human speech as an example of timbres.

4th Lesson: Sound and silence with stories and introducing the concept of intensity through songs and differentiation between utterances.

5th Lesson: Contrast between weak and strong sounds, discrimination between intensities by listening to different musical styles.

6th Lesson: Graphical representation of sound and discrimination of musical styles.

7th Lesson: Introducing the concept of melody and harmony using different instruments and different notes.

### Rhythmic Activities

1st Lesson: Introducing the concept of pulse, body sounds, and discrimination of musical progression through rhythmic variations. In this particular session, firstly, children were asked to mimic the pattern of hand-clapping the teacher was doing. Later, these patterns could vary, and the children had to identify which were the changes and try again to reproduce them according to the teacher’s instructions.

2nd Lesson: Examples of sound duration, discrimination between long and short sounds using musical games, body percussion, and drawings.

3rd Lesson: Explanation of the pulse and musical accent, producing different sounds and rhythms using tongue twisters, poems, and rhymes.

4th Lesson: Concepts of pulse, rhythm, use of different timbres, and rhythms with own body through association with rhythm, movement, and body games.

5th Lesson: Rhythmic association with movements, rhythmic reading through drawings and properties of sounds, and their duration in bodily games and songs.

6th Lesson: Improvising rhythms and accents, the conceptualization of musical breaks through the games of glasses.

7th Lesson: Listening and rhythmic activities, improvising rhythms and accents, differentiation between binary, ternary, and quaternary compasses.

A booklet of the MT was provided for the music teacher with a detailed description of all planned activities per session. Through these guidelines, the teacher could reproduce the same content for each session. Moreover, the music teacher used a guitar to guide participants throughout lessons. To guarantee the understanding during the MT implementation, the researcher and the music teacher had meetings once a week in which the music teacher reported the performance of each child referenced by name and school. This information was not shared with parents or school teachers, i.e., it was exclusively gathered for research quality control.

### Screening Measures

#### Anamnesis (Santos, 2002)

Set of qualitative questions to assess general and specific child development in social, educational, psychological, and health dimensions. It was used as a screening tool for exclusion criteria, as it included questions regarding neurological, psychiatric or psychological disorders, and chronic use of psychoactive substances.

#### SES – Brazilian Association of Marketing Research Institutes Scale (Associação Brasileira de Empresas de Pesquisa [ABEP], 2008)

The Criteria of Economic Classification Brazil is an index consisting of a series of 11 questions about the possession of durable goods and the educational level of the head of the household.

#### Schooling Achievement Test (Stein, 1994)

Set of three subtests: (1) Writing task: In which the experimenter reads out loud a maximum of 34 words and children are required to write them; (2) Reading task: a paper with 70 words is presented to the child who is asked to read; and (3) Arithmetics task: This subtest is composed of two calculation modalities: the first one, contains three oral calculations (for example, “*if you had three sweets and won 4 more, how many would you have now?”);* and the second test included 35 written calculations (from simple calculations, subtraction, multiplication, division to fractions and equations, with one to three digits) in a separate notebook in which children are asked to answer as many questions as they can. Each item presents a range of calculations in ascending order of difficulty, which are presented to children regardless of their school age. The dependent variable for each subtest was the sum of correct answers, and, consequently, the total score was the sum of the three subtests. In the original psychometric study with the SAT, the following Cronbach alpha coefficients were reported: (1) Written task = 0.95; (2) Reading task = 0.99; Arithmetics task = 0.93; and Total score = 0.99 (Stein, 1994).

### Cognitive Measurement

#### Raven’s Colored Progressive Matrices Test (Angelini et al., 1999)

A measure of abstract visual reasoning, which assesses general cognitive ability, in other words, eductive ability (Raven et al., 1998). It is composed of three series with 12 items: A, Ab, and B. In each series, the items are arranged by increasing order of difficulty, each series being more difficult than the previous one. Easier items are always placed at the beginning of each series, which has the purpose of introducing the examinee to a new type of reasoning, which will be required for the following items. The items consist of a drawing or matrix with a missing part. Below the main drawing, six alternatives are presented, one which correctly completes the array. The child must choose one of the alternatives to the missing part. Angelini et al. (1999) presented satisfactory reliability (Cronbach alpha = 0.90).

#### Zareki-R – Battery of Neuropsychological Tests for Number Processing and Calculation in Children-Revised (Von Aster and Dellatolas, 2006)

Zareki-R is an international specialized pencil-and-paper battery test that assesses numerical cognition in school-age children. For this article, subtests were organized into five numerical cognition subtests as described below:

(1) *Number sense –* composed by (i) *Counting dots-* Children have to enumerate different sets of dots. The scoring system considers the number of correct responses and (ii) *Perceptual estimation* – The child has to orally give an estimate of the number of items shown in a picture, which is displayed for 5 s, for example, the number of balls in the picture (the precise answer is 57 balls); (2) *Number Production –* consists of three subtests: (i) *Counting backward* – the participant must count the dots backward, e.g., count the sequences from 23 to 1 and from 67 to 54; (ii) *Dictation of numbers* – the child is asked to write, in arabic numerals, eight numbers orally presented (e.g., 23); and (iii) *Reading Numbers*: The participant should read out loud eight numbers written in arabic numerals, such as 15 and 1900. (3) *Number Comprehension* – is comprised of three subtests: (i) *Oral comparison –* Eight pairs of numbers are verbally presented (e.g., 34601 and 9678) and the child must judge which one is the largest in quantity; (ii) *Contextual estimation*: The child must judge sentences in terms of coherence between quantities and context, for instance: “eight lamps in the same room” is “little,” “median,” or a “lot?”; (iii) *Written comparison*: Pairs of numbers in arabic numeral form are presented visually, for example, 13 and 31, and the child must judge which one is the largest. (4) *Calculation:* includes two subtests (i) *mental calculation*, in which eight additions, eight subtractions and six multiplications are orally presented; and (ii) *Problem solving*: The participant must solve orally presented numerical problems of increasing difficulty. For instance, one of the problems is, “Peter has 12 marbles. He gives 5 to his friend Ann. How many marbles does Peter have now?”. (5) The *Positioning numbers on an analog scale* is a measure of *mental number line*: in this subtest, a vertical line is presented in which the participant is asked to point and mark a specific position indicated by the experimenter. Moreover, the number line task from the ZAREKI-R differs from the classical number line estimation task (Berteletti et al., 2010; Schneider and Siegler, 2010) as the number line in the ZAREKI-R is vertical and not horizontal. For each subtest, the sum of correct answers was calculated, and the total score of Zareki-R is the sum of points in the tests mentioned above, which was used as the dependent variable. The battery also has a measure of phonological memory that is not included in the Zareki-R total score. The *Memory of Digits* requires the forward (FDS) and backward (BDS) repetition of digit sequences of increasing length. Its total score was used as a dependent variable.

The Brazilian normative sample of the Zareki-R (Santos et al., 2012) was formed by 172 children from the same region and cultural backgrounds, which were not attending any training at the time of the study; this sample was used as a normative data set for clinical comparisons. The re-test reliability indicated adequate test-retest reliability (0.87 over a 14-week period) for Zareki-R Total (Ribeiro and Santos, 2017).

### Post-MT Self-Report Evaluation

This questionnaire was designed exclusively for this study to assess if children could identify MT effects through six different dichotomous questions: “After the MT did you: (1) improve your school learning?; (2) notice changes in the way you do math calculations?; (3) improve your grades?; (4) notice changes in your memory?; (5) notice modifications in your mood?; (6) notice alterations in your attention?” (Ribeiro, 2013; Ribeiro and Santos, 2017). Children were asked to answer with *yes* or *no* for each question.

## Statistical Analyses

(1) To investigate the effects of the MT on abstract visual reasoning, memory of digits and numerical cognition performance in DD and TD groups over 14 weeks at four-time points, a series of analyses comparing both groups were performed:
(2) Three separated 2 (Groups: DD and TD) × 4 (measurement time point: baseline, mid-test, post-test, and follow-up) repeated-measures ANOVA were performed for the following dependent variables: abstract visual reasoning percentile, the memory of digits subtest, and Zareki-R Total;
(3) A 2 (Groups: DD, TD) × 4 (measurement time point: baseline, mid-test, post-test, and follow-up) repeated measures MANOVA was performed having as dependent measures numerical cognition systems (number sense, number line, number production, number comprehension, and calculation). All preconditions for conducting ANOVAs and MANOVA were tested (normality and Mauchly’s test of sphericity), and no violation was found.
(4) A non-parametric Mann–Whitney *U* test to compare groups in *Post-MT self-report* evaluation items responses was applied since it included data that were not normally distributed;
(5) We also determined re-test reliabilities at different measurement time points for Zareki-R total.

## Results

### Abstract Visual Reasoning and Memory of Digits

The first 2 (group: DD, TD) × 4 (measurement time point: baseline, mid-test, post-test, and follow-up) repeated measures ANOVA showed no main effect of groups, *F*(1,42) = 1.53, *p* = 0.22, *MSE* = 1149.53, η*^2^_*p*_* = 0.03, or time, *F*(3,126) = 1.57, *p* = 0.20, *MSE* = 106.81, η*^2^_*p*_* = 0.04. In addition, no interaction effect was found between groups and time points regarding the percentile of abstract visual reasoning scores, *F*(3,126) = 0.09, *p* = 0.97; *MSE* = 106.81, η*^2^_*p*_* = 0.002. Mean percentile ranks and standard deviations of abstract visual reasoning scores are displayed in Table 2. Concerning the 2 × 4 repeated-measure ANOVA conducted for the memory of digits subtest, a time effect was found, *F*(3,126) = 5.47, *p* = 0.001, *MSE* = 15.18, η*^2^_*p*_* = 0.11, in which, according to pairwise comparisons, baseline had lower scores when compared to post-test (*p* = 0.005) and follow-up times (*p* = 0.006). However, no group effect, *F*(1,42) = 2.19, *p* = 0.14, *MSE* = 74.92, η*^2^_*p*_* = 0.05, or interaction between groups and time points was observed, *F*(3,126) = 0.85, *p* = 0.47, *MSE* = 15.18, η*^2^_*p*_* = 0.02.

### Numerical Cognition

The following ANOVA results for Zareki-R total revealed a main effect of group, *F*(1,42) = 46.04, *p* < 0.001, *MSE* = 710.86, η*^2^_*p*_* = 0.52. Pairwise comparisons revealed that the DD group had worse performance in the Zareki-R total score compared to the TD group (*p* = 0.001). A main effect of time was also verified, *F*(3,126) = 61.33, *p* < 0.001, *MSE* = 105.40, η*^2^_*p*_* = 0.59, in which comparisons showed that baseline results were lower as compared to mid-test, post-test, and follow-up test results (*p*s < 0.001). Finally, there was a significant interaction effect between group and times, *F*(3,126) = 10.97, *p* < 0.001, *MSE* = 105.40, η*^2^_*p*_* = 0.21. These interaction shows the effects of MT on the Zareki-R total changes according to groups.

To see whether groups differed with regard to numerical cognition abilities, we carried out the 2 (Groups: DD, TD) × 4 (measurement time point: baseline, mid-test, post-test, and follow-up) repeated measures MANOVA. The results showed a significant group effect, *F*(1,42) = 43.96, *p* < 0.001, *MSE* = 32.97, η*^2^_*p*_* = 0.51, in which the DD group showed lower scores than the TD group (*p* < 0.001).

To disentangle this significant interaction, we performed separate repeated measures ANOVAs for the DD and TD groups. Results indicated significant effects only for the DD group on number production- [*F*(3,63) = 36.54, *p* < 0.001, η*^2^_*p*_* = 0.63], in which pairwise comparisons showed that baseline score was lower compared to the other three assessed timepoints (*p*s < 0.001) and mid-test performance was lower than follow-up performance.

**FIGURE 2:**
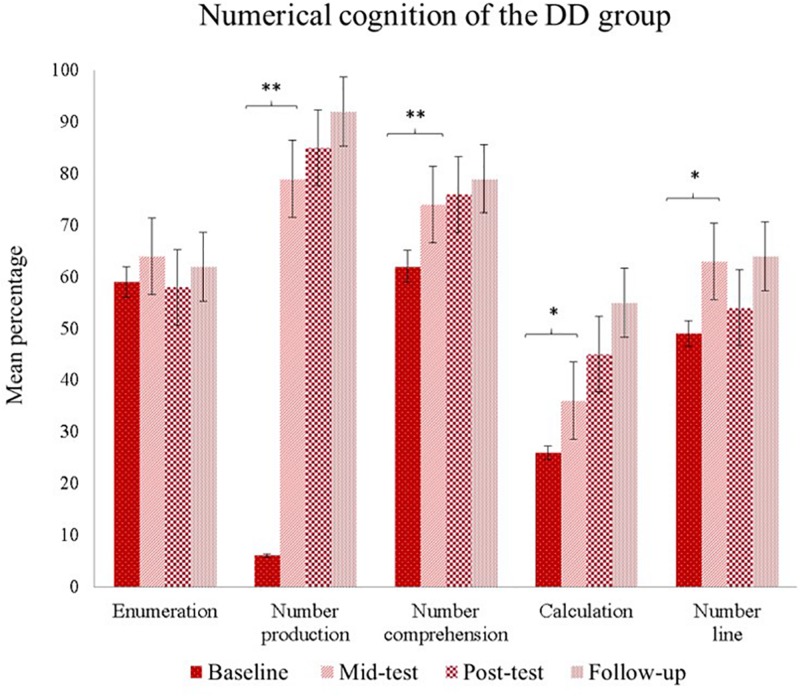
Pairwise *post hoc* tests among different times of the DD group percentage scores and standard error. *^∗^p* < 0.05; ^∗∗^*p* < 0.01.

For number comprehension – [*F*(3,63) = 18.00, *p* < 0.001, η*^2^_*p*_* = 0.46], baseline performance was lower as compared to the other assessed timepoints (*p*s < 0.002); Calculation – [*F*(3,63) = 21.72, *p* < 0.001, η*^2^_*p*_* = 0.51], baseline was lower compared to the other assessment times (*p*s < 0.02) and Mid-test performance was poorer compared to the follow-up (p < 0.001) (Figure 2). We displayed in Table 3 mean and standard deviations of raw scores and the percentage of correct responses for numerical cognition systems in the four timepoints.

**TABLE 3:** Mean percentile (and standard deviation) of CPM, raw scores (and standard deviation) and percentage of correct answers on numerical cognition assessment for DD and TD groups at four-time points (baseline to follow-up).

	**DD (*n* = 22)**										**TD (*n* = 22)**						
		
**CPM**	**Baseline**	**%**	**Mid-test**	**%**	**Post-test**	**%**	**Follow-up**	**%**	**Baseline**	**%**	**Mid-test**	**%**	**Post-test**	**%**	**Follow-up**	**%**	**Max**
Percentile	69.27 (18.13)		71.54 (22.64)		70.45 (22.35)		73.14 (21.01)		74.54 (15.27)		79.04 (18.18)		76.59 (14.75)		79.54 (19.39)		
**Numerical cognition**																	
Enumeration	4.16 (1.22)	17	4.48 (1.55)	19	4.09 (1.49)	17	4.32 (1.18)	18	5.32 (1.16)	22	5.07 (1.07)	21	4.57 (1.68)	19	5.02 (1.18)	21	24
Number production	7.20 (2.29)	20	9.53 (1.87)	26	10.16 (1.77)	28	11.02 (0.77)	31	10.17 (1.54)	28	10.59 (1.05)	29	10.76 (2.32)	30	11.46 (0.50)	32	36
Number comprehension	11.63 (2.06)	25	13.76 (2.07)	30	14.12 (2.40)	31	14.73 (2.20)	32	14.58 (1.64)	32	15.55 (1.38)	34	15.25 (3.56)	33	16.55 (1.08)	36	46
Calculation	7.27 (4.13)	13	10.18 (5.01)	18	12.57 (5.22)	22	15.43 (5.89)	28	16.23 (3.90)	29	17.91 (4.55)	32	18.34 (6.30)	33	19.25 (4.94)	34	56
Number line	11.82 (4.80)	49	15.05 (3.47)	63	13.07 (3.63)	54	15.02 (3.38)	64	14.75 (3.70)	61	15.77 (3.53)	66	14.52 (4.53)	61	16.59 (2.74)	69	24
Memory of digits	21.64 (4.77)	45	24.09 (5.36)	50	24.09 (5.29)	50	25.45 (4.83)	53	24.09 (5.87)	50	25.36 (5.67)	53	27.27 (5.81)	57	26.27 (6.15)	55	48
Zareki-R total	91.18 (13.26)	49	114.23 (17.59)	61	119.20 (21.41)	64	132.00 (19.24)	71	132.48 (13.38)	71	140.14 (12.92)	75	144.43 (15.55)	78	149.14 (12.36)	80	186

*CPM, Raven’s colored progressive matrices; SD, standard deviation; DD, children with developmental dyscalculia; TD, typical development children which underwent MT; *n*, number of participants;%, percentage of correct responses for each numerical cognition; Max, *highest possible raw score.**

**FIGURE 3:**
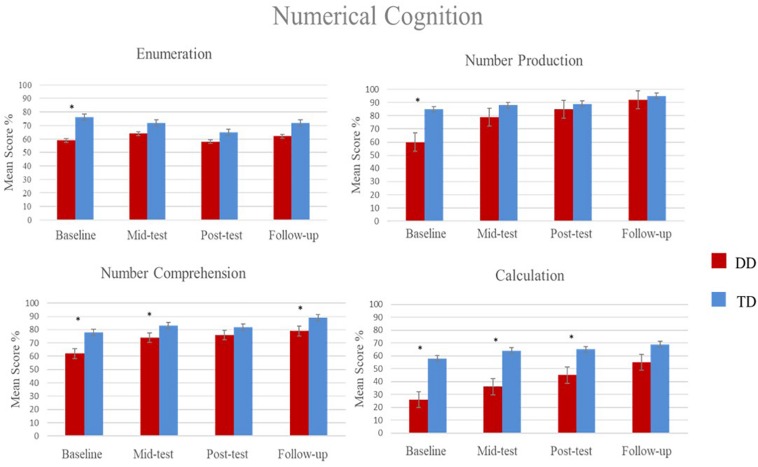
Mean percentage of correct responses and standard error on numerical cognition components, comparing the DD group, and the TD group. *^∗^p*s < 0.012.

In order to compare group performance on numerical cognition, the data was analyzed via a t-test for independent samples for each numerical cognition system with a Bonferroni correction for multiple tests resulting in a significance level of *p* = 0.012 (*p* = 0.05/4 – four timepoints). Results revealed higher scores for TD group on number sense at Baseline, *t*(42) = −3.23, *p* = 0.002; On number production at Baseline, *t*(42) = −5.07, *p* < 0.001; On number comprehension at Baseline, *t*(42) = −5.25, *p* < 0.001, mid-test, *t*(42) = −3.36, *p* = 0.002 and follow-up, *t*(42) = −3.49, *p* < 0.001; On Calculation at Baseline; *t*(42) = −7.39, *p* < 0.001; mid-test, *t*(42) = −5.36; *p* < 0.001; and post-test, *t*(42) = −3.31; *p* < 0.01; Significant statistical results are shown in Figure 3.

### Post-MT Self-Report Evaluation

**FIGURE 4:**
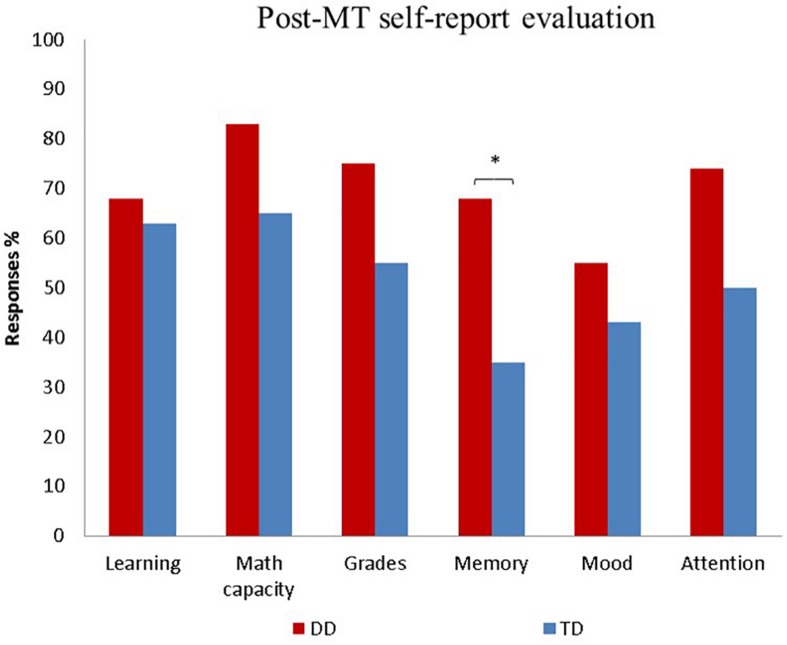
Response percentage of the DD and the TD groups on the Post-MT self-report evaluation. ^∗^*p* < 0.05.

To investigate whether groups differed in the self-report evaluation of learning, math learning, grades, memory, mood, and attention after the MT, we carried out six separate Mann–Whitney *U* tests. Results showed significant results just for self-reported memory capacity, *U* = 143, *p* = 0.04, in which DD group showed more yes responses to higher memory capacity after the MT compared to the TD group. The percentage of children in each group who said *yes* on each of the questions is displayed in Figure 4.

### Test-Retest Reliability

The test-retest reliability was conducted to investigate the Zareki-R temporal stability. The test-retest reliability results using Pearson’s test detected strong positive and significant correlations between the test and retest for Zareki-R (Baseline – mid-test = 0.87, mid-test – post-test = 0.87, and post-test – follow-up = 0.87).

## Discussion

In this longitudinal, double-blind, and pseudo-randomized study with mixed design, the between-groups comparison tested for effects of the MT in children with DD compared to children with TD in measures of numerical cognition and abstract visual reasoning, while the within-group comparison tested changes at specific time points. Far transfer effects due to MT stimulation were investigated comparing both the DD and the TD groups at four assessments: Baseline, mid-test, post-test at the end of MT, and a Follow-up 10 months after the end of MT.

As far as we know, there are no other longitudinal studies with a follow-up currently available to contrast the efficacy of organic MT activities on numerical cognition and visual abstract reasoning nor data showing its effectiveness. Importantly, this study was carried out in a clinical sample, mostly understudied employing a proper operational diagnosis supported by psychometric measures and the medical manuals. In terms of epidemiology, our study followed a two-phase diagnostic assessment, i.e., the first phase comprised the screening of intelligence and schooling achievement, such as writing, arithmetics, and reading abilities and the second phase was the diagnosis (ICD-10, F81.2; World Health Organization [WHO], 2018) confirmation based on the Zareki-R’s cut-off criteria (Rotzer et al., 2009), with a validated battery for Brazilian children that allowed an accurate diagnosis. We found a similar prevalence rate to international studies (Butterworth, 2005; Shalev et al., 2005; Von Aster et al., 2007; Sigmundsson et al., 2010) and national ones that showed higher prevalence in boys with DD than girls (Bastos et al., 2016; Fortes et al., 2016). Moreover, our sample apparently had a rare phenotype of primary DD, i.e., children neither had comorbidities, nor other cognitive deficits apart from the ones in numerical cognition (von Aster and Shalev, 2007; Ashkenazi and Henik, 2010; Kaufmann et al., 2013; Ribeiro et al., 2017). The comparison between groups indicated that the DD group had lower scores at the baseline in many numerical abilities (Number sense, number production, number comprehension, and calculation) compared to the TD group. Clinically, i.e., considering normative data (Santos et al., 2012), only number line performance corresponded to the expected age mean score on the posttest, and the overall low performance confirms the diagnosis of DD (Rotzer et al., 2009).

Congruently to our first hypothesis, the longitudinal perspective revealed that children with DD that accomplished the MT outperformed themselves from the baseline to mid-test in number production, number comprehension, number line, and from the baseline to follow up in calculation. This result suggests that the DD group was more responsive to MT since they had room to grow in performance. By contrast, the TD group neither shows statistical nor clinical changes, since their mean scores were already equivalent to their age counterparts (Santos et al., 2012). This result is congruent with studies showing that MT could cause modest or null far-transfer in children with TD (Sala and Gobet, 2017).

Furthermore, it is essential to make clear that children in the DD and TD groups did not receive any additional training during baseline until post-test, and that formal math instruction at school was the same for all of them; only in the follow-up two of these children had started music classes unrelated to MT. Apart from that, changes do not seem to be due to successive assessments with the same tasks, i.e., learning effects. If this was the case, the retest-reliability would be lower (Spreen and Strauss, 1998), and performances in the tasks should be consistently better over time for both trained groups, which was not detected.

Observing numerical systems individually in children with DD, the data revealed improvements until 10 months after the post-test, which might be named as remediation. At least for 10 months after the post-test, children with DD retained gains in the number comprehension and the number line tasks. Changes in number line task after intervention are assumed as a core improvement in numerical cognition *per se* and associated with brain changes (Kucian et al., 2011). A plausible explanation for this result would be that the MT activities did facilitate symbolic representations (Graziano et al., 1999; Vaughn, 2000; Gromko, 2004; Schlaug et al., 2005; Nutley et al., 2014) when children were given tasks such as drawing the size or the tone of the sound.

Additionally, in the follow-up, the TD group outperformed the DD group in number production; even though based on normative data from the Zareki-R validation sample (Santos et al., 2012), the groups were equivalent. The calculation was the only ability that remained below average at the last time point, in defiance of slightly increased scores in the course of repeated assessments. The lower results in the calculation were influenced by the abilities to perform mental calculation and problem-solving tasks orally since the sum of these scores comprised the calculation system.

The resistance of these abilities to intervention across time is well established (Shalev et al., 2005; Mazzocco and Räsänen, 2013) and suggests that the DD children remain unable to follow the steps required to effectively perform the calculation (McCloskey et al., 1985; Skagerlund and Träff, 2016). However, bearing in mind the increasing trajectory of calculation after MT, perhaps remediation could be achieved with longer intervention. As would be expected in this neurodevelopmental disorder, the Zareki-R Total of the DD group is still lower than the TD group scores at this time point due to calculation deficits.

In the case of the number line, results indicated that this core ability of numerical cognition frequently impaired in children with DD is responsive to MT stimulation. Although the task we used differs from the classical number line estimation (c.f., horizontal one as Berteletti et al., 2010; Schneider and Siegler, 2010), it has been previously studied in computer assistance intervention. Successful performance in the vertical number line task is associated with comprehension of the connection between numerical magnitudes, ordinality, and precise number representation (Kucian et al., 2011; Käser et al., 2013) and we infer that MT stimulates this connection in children with DD (Michels et al., 2018).

These outcomes indicate that MT provided a fruitful basis for children with DD to access the symbolic numbers’ magnitude representation enabling learning in regular math classes, which conceivably corroborates, to some extent, the studies which used instrumental MT for number production and comprehension (Graziano et al., 1999; Vaughn, 2000; Gromko, 2004; Schlaug et al., 2005; Nutley et al., 2014).

As far as we know, this was the first study that used the MT as a remediation tool for children with DD, and the outcome suggests that even complex abilities, such as calculation, may improve with this kind of training. Despite the lack of untrained control groups, we can partially assume that our MT would be beneficial to mathematical knowledge because it produced far transfer effects in numerical cognition after controlling several variables that could influence groups performances. By contrast, regular math classes, and even conventional educational interventions, apparently might not cause significant changes in numerical cognition capacity according to follow up studies (Shalev et al., 1998, 2005; Mazzocco and Räsänen, 2013). Moreover, *the Post-MT self-report evaluation* indicated that children with DD were aware of their improvement in math and 60 to 80% of trained children spontaneously and explicitly associated math gains to MT.

On the baseline, the DD group performed abstract visual reasoning in equivalence to the TD group. There was no significant interaction between measurement timepoint and tested groups, revealing that MT did not produce a far transfer effect to the abstract visual reasoning, corroborating Sala and Gobet (2017). As previously demonstrated by Schellenberg (2004), the impact on IQ is relatively small regardless whether researchers use IQ subtests or the sum of the scores. Being so, abstract visual reasoning seems to be a particular task, which could not be influenced by MT. Nevertheless, differences in MT activities should be further studied to make clear on which aspects of cognition MT has a beneficial effect.

The far transfer effects for numerical cognition described here were confirmed by cluster analyses contrasting two-time points in a previous study (Ribeiro and Santos, 2017). The number of children assigned to the control group was larger on the post-test, i.e., eight children with DD normalized their math scores after the MT (Ribeiro and Santos, 2017).

The outcomes of this study indicate that children having mathematics difficulties could benefit from our organic MT to improve learning. At the same time, the organic MT motivated children barely familiar with music as art toward instrumental music education. Instrumental music teachers that are frequently involved in group music teaching and teaching in various educational contexts could incorporate some of these elements in their practice. For instance, the instrumental music teacher should teach apart from the technical aspects of playing a musical instrument, they should promote motivation to children express themselves through music, develop aural skills and ability to children be musically inventive which later on might result in interest to pursue instrumental music learning.

Furthermore, in our view, our organic MT is a pleasant technique that has the advantage of being accomplished at school and in mixed groups, which favors inclusion and socialization, in addition, it does not require mobility by parents and students, because the activities could be carried out at the school, requiring only an educator with formal musical education.

### Strengths, Limitations, and Perspectives

This study describes the basic structure of an MT program and its effects in order to expand knowledge about the techniques that are suitable for cognitive remediation. Also, the protocol had specific tasks for different components of numerical cognition, providing a scrutinized view of these abilities.

One of the limitations of this study was the design, which did not include untrained DD and TD groups throughout the four-time points. It was not feasible since the screening phase, MT, and all assessments must occur while the child remains in the same school year. Otherwise, the cognitive scores, which are age- or schooling-related, would not be suitable for comparison with the baseline, even though we cannot ignore that the math content and practice might increase across the school year as the general development.

Considering the prevalence rate of DD, we should search for a new cohort of around 400 children to find another sample of 22 children. Moreover, this design was preferred due to ethical constraints since the ethics committee would not approve a research project having an untrained DD group. Nevertheless, our sample was considerably larger than in other studies with a similar design (Kucian et al., 2011; Ashkenazi and Henik, 2012; Michels et al., 2018). Furthermore, we controlled math classes on formal education to be similar across schools and also requested information regarding extracurricular courses confirming that children did not engage in extra activities besides the MT. Apart from that, we had normative data to contrast performances from the Zareki-R validation carried out by Santos et al. (2012), which, in a broad sense, are “untrained” children.

Although we applied rhythmic and melodic lessons, it was not our aim to investigate which of the two activities were most effective in numerical cognition. It would be important that researchers could systematize the findings of diverse MT strategies. However, another type of design should be applied, such as a crossover study to investigate each of the methods, for instance, an organic MT vs. a formal instrumental MT.

As for future directions, it is essential to replicate MT in other samples with DD and to explore its effects in other learning disabilities, aiming to understand possible transfers and neuroplasticity. Moreover, future studies may explore ecological transfer measures, such as children’s school grades across the school year, and also specific musical abilities, e.g., rhythmic and melodic, which can later be used for cognitive remediation of different types of learning disabilities, if possible, with neuroimaging techniques. In line with these considerations, future research will be essential to explore the efficiency and cost-effectiveness of such methods.

### Conclusion

In conclusion, the DD group showed slight improvements in numerical cognition throughout 14 sessions of a brief MT, especially for number production, number comprehension, and calculation. Scores for calculation remained better for the DD group compared with their baseline, but lower compared to the TD group performance throughout the training, which shows that the calculation deficits seem to be longer-lasting in the DD group. On the other hand, the follow up indicated that the MT benefits in numerical cognition remained at least 10 months after training. Moreover, the organic MT seems to produce beneficial cognitive effects similar to those obtained with instrumental MT with the advantage of being appropriate for environments with socioeconomic disadvantage.

## Data Availability Statement

The datasets generated for this study are available on request to the corresponding author.

## Ethics Statement

The studies involving human participants were reviewed and approved by the Ethics committee of UNESP, São Paulo State University. Written informed consent to participate in this study was provided by the participants’ legal guardian/next of kin.

## Author Contributions

FS designed the study. FR developed the intervention programme, analyzed the data and drafted the initial manuscript. Both authors contributed to the interpretation of the results, revised the manuscript, and approved its final version.

## Conflict of Interest

The authors declare that the research was conducted in the absence of any commercial or financial relationships that could be construed as a potential conflict of interest.

## References

[B1] Ancker J. S. Kaufman D. (2007). Rethinking health numeracy: a multidisciplinary literature review. *JAMIA* 14 713–721. 10.1197/jamia.M2464 17712082 PMC2213486

[B2] Angelini A. L. Alves I. C. B. Custódio E. M. Duarte W. F. Duarte J. L. M. (1999). *Matrizes Progressivas Coloridas de Raven: Escala Especial. Manual.* São Paulo: CETEPP.

[B3] Ashkenazi S. Henik A. (2010). Attentional networks in developmental dyscalculia. *Behav. Brain Funct.* 6:2. 10.1186/1744-9081-6-2 20157427 PMC2821357

[B4] Ashkenazi S. Henik A. (2012). Does attentional training improve numerical processing in developmental dyscalculia? *Neuropsychology* 26:45. 10.1037/a0026209 22081984

[B5] Associação Brasileira de Empresas de Pesquisa [ABEP], (2008). *Critério de Classificação Econômica no Brasil.* Available at: http://www.abep.org/criterio-brasil (accessed October 5, 2013).

[B6] Arias-Rodriguez I. Mendes do Nascimento J. Voigt M. F. Dos Santos F. H. (2019). Numeracy musical training for school children with low achievement in mathematics. *Ann. Psychol.* 35 405–416. 10.6018/analesps.35.3.340091

[B7] Azaryahu L. Courey S. J. Elkoshi R. Adi-Japha E. (2019). ‘MusiMath’ and ‘Academic Music’ – Two music-based intervention programs for fractions learning in fourth grade students. *Devel. Sci.* e12882. 10.1111/desc.12882 31250477 PMC7378943

[B8] Bastos J. A. Cecato A. M. T. Martins M. R. I. Grecca K. R. R. Pierini R. (2016). The prevalence of developmental dyscalculia in Brazilian public school system. *Arquivos de Neuro-Psiquiatria* 74 201–206. 10.1590/0004-282X20150212 27050848

[B9] Berteletti I. Lucangeli D. Piazza M. Dehaene S. Zorzi M. (2010). Numerical estimation in preschoolers. *Dev. Psychol.* 46 545–551. 10.1037/a0017887 20210512

[B10] Butterworth B. (2005). The development of arithmetical abilities. *J. Child Psychol. Psychiatry* 46 3–18. 10.1111/j.1469-7610.2004.00374.x 15660640

[B11] Castro M. V. Bissaco M. A. S. Panccioni B. M. Rodrigues S. C. M. Domingues A. M. (2014). Effect of a virtual environment on the development of mathematical skills in children with dyscalculia. *PLoS One* 9:e0103354. 10.1371/journal.pone.0103354 25068511 PMC4113388

[B12] Cheng D. Xiao Q. Cui J. Chen C. Zeng J. Chen Q. (2019). Short-term numerosity training promotes symbolic arithmetic in children with developmental dyscalculia: the mediating role of visual form perception. *Dev. Sci.* e12910. 10.1111/desc.12910 31599035

[B13] Clark A. Chalmers D. (1998). The extended mind. *Analysis* 58 7–19. 10.1111/1467-8284.00096

[B14] Davidson R. J. McEwen B. S. (2012). Social influences on neuroplasticity: stress and interventions to promote well-being. *Nat. Neurosci.* 15 689–695. 10.1038/nn.3093 22534579 PMC3491815

[B15] Dehaene S. (1997). *The Number Sense.* New York, NY: Oxford University Press.

[B16] Dehaene S. Bossini S. Giraux P. (1993). The mental representation of parity and number magnitude. *J. Exp. Psychol.* 122 371–396. 10.1037/0096-3445.122.3.371

[B17] Devine A. Soltész F. Nobes A. Goswami U. Szücs D. (2013). Gender differences in developmental dyscalculia depend on diagnostic criteria. *Learn. Instruc.* 27 31–39. 10.1016/j.learninstruc.2013.02.004 27667904 PMC4461157

[B18] Donald M. (1991). *Origin of the Modern Mind:Three Stages in the Evolution of Culture and Cognition.* Cambridge, MA: Harvard University Press.

[B19] Dumont E. Syurina E. V. Feron F. J. M. van Hooren S. (2017). Music interventions and child development: a critical review and further directions. *Front. Psychol.* 8:1694. 10.3389/fpsyg.2017.01694 29033877 PMC5626863

[B20] Elmer S. Hänggi J. Jäncke L. (2016). Interhemispheric transcallosal connectivity between the left and right planum temporale predicts musicianship, performance in temporal speech processing, and functional specialization. *Brain Struct. Funct.* 221 331–344. 10.1007/s00429-014-0910-x 25413573

[B21] Elofsson J. Gustafson S. Samuelsson J. Träff U. (2016). Playing number board games supports 5-year-old children’s early mathematical development. *J. Math. Behav.* 43 134–147. 10.1016/j.jmathb.2016.07.003

[B22] Esteki M. (2013). Effectiveness of “Music Training” on reorganization of brain and poor intellectual abilities in female students with dyscalculia (7-9 years old). *Glob. J. Arts Educ.* 3 06–10.

[B23] Fingelkurts A. A. Fingelkurts A. A. Kähkönen S. (2005). Functional connectivity in the brain—is it an elusive concept? *Neurosci. Biobehav. Rev.* 28 827–836. 10.1016/j.neubiorev.2004.10.009 15642624

[B24] Fortes I. S. Paula C. S. Oliveira M. C. Jesus-Mari I. A. B. Rohde L. A. (2016). A cross-sectional study to assess the prevalence of DSM-5 specific learning disorders in representative school samples from the second to sixth grade in Brazil. *Eur. Child Adolesc. Psychiatry* 25 195–207. 10.1007/s00787-015-0708-2 25925785

[B25] Fuchs L. S. Geary D. C. Compton D. L. Fuchs D. Schatschneider C. Hamlett C. L. (2013). Effects of first-grade number knowledge tutoring with contrasting forms of practice. *J. Educ. Psychol.* 105 58–77. 10.1037/a0030127 24065865 PMC3779611

[B26] Gallagher S. (2005). *How the Body Shapes the Mind.* New York, NY: Oxford University Press.

[B27] Geary D. C. (1993). Mathematical disabilities: cognitive, neuropsychological, and genetics components. *Psychol. Bull.* 114 345–362. 10.1016/j.lindif.2009.10.008 8416036

[B28] Goulart D. (2000). *Dalcroze, Orff, Suzuki e Kodály: Semelhanças, Diferenças, Especificidades.* Rio de Janeiro: Conservatório Brasileiro de Música do Rio de Janeiro.

[B29] Graziano A. B. Peterson M. Shaw G. L. (1999). Enhanced learning of proportional math through music training and spatial- temporal training. *Neurol. Res.* 21 139–152. 10.1080/01616412.1999.11740910 10100200

[B30] Gromko J. E. (2004). Predictors of music sight-reading ability in high school wind players. *J. Res. Music Educ.* 52 6–15. 10.2307/3345521

[B31] Guhn M. Emerson S. D. Gouzouasis P. (2019). A population-level analysis of associations between school music participation and academic achievement. *J. Educ. Psychol.* 10.1037/edu0000376

[B32] Hudziak J. J. Albaugh M. D. Ducharme S. Karama S. Spottswood M. Crehan E. (2014). Cortical thickness maturation and duration of music training: health-promoting activities shape brain development. *J. Am. Acad. Child Adolesc. Psychiatry* 53 1153–1161. 10.1016/j.jaac.2014.06.015 25440305 PMC4254594

[B33] INEP (2009). *‘Censo escolar’, Instituto Nacional de Estudos e Pesquisas Educacionais Anísio Teixeira.* Available at: http://portal.inep.gov.br/web/guest/educacao-basica (accessed November 15, 2010).

[B34] Iuculano T. Roserberg-Lee M. Richardson J. Tenison C. Fuchs L. Superkar K. (2015). Cognitive tutoring induces widespread neuroplasticity and remediates brain function in children with mathematical learning disabilities. *Nat. Commun.* 6:8453. 10.1038/ncomms9453 26419418 PMC4598717

[B35] James C. E. Oechslin M. S. Michel C. M. De Pretto M. (2017). Electrical neuroimaging of music processing reveals mid-latency changes with level of musical expertise. *Front. Neurosci.* 11:613. 10.3389/fnins.2017.00613 29163017 PMC5682036

[B36] Käser T. Baschera G.-M. Kohn J. Kucian K. Richtmann V. Grond U. (2013). Design and evaluation of the computer-based training program Calcularis for enhancing numerical cognition. *Front. Psychol.* 4:489. 10.3389/fpsyg.2013.00489 23935586 PMC3733013

[B37] Kaufmann L. Mazzocco M. M. Dowker A. Von Aster M. Göbel S. M. Nuerk H. (2013). Dyscalculia from a developmental and differential perspective. *Front. Psychol.* 4:516. 10.3389/fpsyg.2013.00516 23970870 PMC3748433

[B38] Kaufmann L. Wood G. Rubinsten O. Henik A. (2011). Meta-analysis of developmental fMRI studies investigating typical and atypical trajectories of number processing and calculation. *Dev. Neuropsychol.* 36 763–787. 10.1080/87565641.2010.549884 21761997

[B39] Koelsch S. (2010). Towards a neural basis of music-evoked emotions. *Trends Cogn. Sci.* 14 131–137. 10.1016/j.tics.2010.01.002 20153242

[B40] Koelsch S. E. Siebel W. A. (2005). Towards a neural basis of music perception. *Trends Cogn. Sci.* 9 578–584. 10.1016/j.tics.2005.10.001 16271503

[B41] Kroeger L. A. Brown R. D. O’Brien B. A. (2012). Connecting neuroscience, cognitive, and educational theories and research to practice: a review of mathematics intervention programs. *Early Educ. Dev.* 23 37–58. 10.1080/10409289.2012.617289

[B42] Krueger J. (2018). “Music as affective scaffolding,” in *Music and Consciousness II*, eds Clarke D. Herbert R. Clarke E. (Oxford: Oxford University Press).

[B43] Kucian K. Grond U. Rotzer S. Henzi B. Schönmann C. Plangger F. (2011). Mental number line training in children with developmental dyscalculia. *Neuroimage* 57 782–795. 10.1111/j.1749-6632.2011.06439.x 21295145

[B44] Kucian K. Von Aster M. (2015). Developmental dyscalculia. *Eur. J. Pediatrics* 174 1–13. 10.100/s00431-014-2455-7 25529864

[B45] Landerl K. (2013). Development of numerical processing in children with typical and dyscalculic arithmetic skills—a longitudinal study. *Front. Psychol.* 4:459. 10.3389/fpsyg.2013.00459 23898310 PMC3720999

[B46] Mazzocco M. M. M. Räsänen P. (2013). Contributions of longitudinal studies to evolving definitions and knowledge of developmental dyscalculia. *Trends Neurosci. Educ.* 2 65–73. 10.1016/j.tine.2013.05.001

[B47] McCloskey M. Caramazza A. Basili A. (1985). Cognitive mechanisms in number processing and calculation: evidence from dyscalculia. *Brain Cogn.* 4 171–196. 10.1016/0278-2626(85)90069-7 2409994

[B48] McIntosh A. R. (2000). Towards a network theory of cognition. *Neural Netw.* 13 861–870. 10.1016/s0893-6080(00)00059-9 11156197

[B49] Michels L. O’Gorman R. Kucian K. (2018). Functional hyperconnectivity vanishes in children with developmental dyscalculia after numerical intervention. *Dev. Cogn. Neurosci.* 30 291–303. 10.1016/j.dcn.2017.03.005 28442224 PMC6969091

[B50] Miendlarzewska E. A. Trost W. J. (2013). How musical training affects cognitive development: rhythm, reward and other modulating variables. *Front. Neurosci.* 7:279. 10.3389/fnins.2013.00279 24672420 PMC3957486

[B51] Monei T. Pedro A. (2017). A systematic review of interventions for children presenting with dyscalculia in primary schools. *Educ. Psychol. Pract.* 33 277–293. 10.1080/02667363.2017.1289076

[B52] Moore E. Schaefer R. S. Bastin M. E. Roberts N. Overy K. (2014). Can musical training influence brain connectivity? Evidence from diffusion tensor MRI. *Brain Sci.* 4 405–427. 10.3390/brainsci4020405 24961769 PMC4101485

[B53] Nutley S. B. Darki F. Klingberg T. (2014). Music practice is associated with development of working memory during childhood and adolescence. *Front. Hum. Neurosci.* 7:926. 10.3389/fnhum.2013.00926 24431997 PMC3882720

[B54] OECD, (2015). *OECD Environmental Performance Reviews: Brazil 2015.* Paris: OECD Publishing, 10.1787/9789264240094-en

[B55] Oechslin M. S. Van De Ville D. Lazeyras F. Hauert C. A. James C. E. (2013). Degree of musical expertise modulates higher order brain functioning. *Cereb. Cortex* 23 2213–2224. 10.1093/cercor/bhs206 22832388

[B56] Rauscher F. H. Shaw G. L. Levine L. J. Ky K. N. Wright E. L. (1994). “Music and spatial task performance: A causal relationship,” *Paper Presented at the American Psychological Association 102nd Annual Convention*, Los Angeles, CA.

[B57] Raven J. Raven J. C. Court J. H. (1998). *updated 2004). Manual for Raven’s Progressive Matrices and Vocabulary Scales. Sections 1-7 with 3 Research Supplements.* San Antonio, TX: Harcourt Assessment.

[B58] Ribeiro F. S. (2013). *O efeito do treino musical sobre a capacidade da memória operacional e da cognição numérica de crianças com discalculia do desenvolvimento.* Master’s thesis, Universidade Estadual Paulista - Júlio de Mesquista Filho, Bauru

[B59] Ribeiro F. S. Santos F. H. (2012). Treino musical e capacidade da memória operacional em crianças iniciantes, veteranas e sem conhecimentos musicais. *Psicologia Reflexão e Crítica* 25 559–567. 10.1590/S0102-79722012000300016

[B60] Ribeiro F. S. Santos F. H. (2015). “Métodos específicos para impulsionar a memória operacional,” in *Neuropsicologia Hoje*, eds Santos F. H. Andrade V. M. Bueno O. F. A. (São Paulo: Artmed), 299–306.

[B61] Ribeiro F. S. Santos F. H. (2017). Enhancement of numeric cognition in children with numeracy deficits after a non instrumental musical training. *Res. Dev. Disabil.* 62 26–39. 10.1016/j.ridd.2016.11.008 28107681

[B62] Ribeiro F. S. Tonoli M. C. Ribeiro D. P. S. A. Santos F. H. (2017). Numeracy deficits scrutinized: evidences of primary developmental dyscalculia. *Psychol. Neurosci.* 10 189–200. 10.1037/pne0000082

[B63] Ritchie S. J. Bates T. C. (2013). Enduring links from childhood mathematics and reading achievement to adult socioeconomic status. *Psychol. Sci.* 24 1301–1308. 10.1177/0956797612466268 23640065

[B64] Rotzer S. Loenneker T. Kucia K. Martin E. Klaver P. Von Aster M. (2009). Dysfunctional neural network of spatial working memory contributes to developmental dyscalculia. *Neuropsychologia* 47 2859–2865. 10.1016/j.neuropsychologia.2009.06.009 19540861

[B65] Ryan K. Schiavio A. (2019). Extended Musicking, extended mind, extended agency. Notes on the third wave. *New Ideas Psychol.* 55 8–17. 10.1016/j.newideapsych.2019.03.001

[B66] Sala G. Gobet F. (2017). When the music’s over. Does music skill transfer to children’s and young adolescents’ cognitive and academic skills? A meta-analysis. *Educ. Res. Rev.* 20 55–67. 10.1016/j.edurev.2016.11.005

[B67] Sanders E. M. (2012). Investigating the relationship between musical training and mathematical thinking in children. *Proc. Soc. Behav. Sci.* 55 1134–1143. 10.1016/j.sbspro.2012.09.607

[B68] Sanders E. M. (2018). *Music Learning and Mathematics Achievement: A Real-World Study.* Doctoral thesis, English Primary Schools, Geneva 10.17863/CAM.30977.

[B69] Santos F. H. (2002). *Memória Operacional de Crianças Normais e com Lesões Congênitas: Desenvolvimento Cognitivo e Reorganização Cerebral.* Unpublished Doctoral disseration Universidade Federal de São Paulo, São Paulo, SP.

[B70] Santos F. H. Silva P. A. Ribeiro F. S. Dias A. L. R. P. Frigério M. C. Dellatolas G. (2012). Number processing and calculation in brazilian children aged 7-12 years. *Span. J. Psychol.* 15 513–525. 10.5209/rev_SJOP.2012.v15.n2.38862 22774425

[B71] Schellenberg E. G. (2004). Music lessons enhance IQ. *Psychol. Sci.* 15 511–514. 10.1111/j.0956-7976.2004.00711.x 15270994

[B72] Schellenberg E. G. (2019). Correlation = Causation? Music training, psychology, and neuroscience. *Psychol. Aesthet. Creat. Arts* 10.1037/aca0000263

[B73] Schellenberg E. G. Weiss M. W. (2013). “Music and cognitive abilities,” in *The Psychology of Music*, 3rd Edn, ed. Deutsch D. (Amsterdam: Elsevier), 499–550. 10.1016/B978-0-12-381460-9.00012-2

[B74] Schiavio A. Altenmüller E. (2015). Exploring music-based rehabilitation for Parkinsonism through embodied cognitive science. *Front. Neurol.* 6:217. 10.3389/fneur.2015.00217 26539155 PMC4609849

[B75] Schiavio A. van der Schyff D. (2018). 4E music pedagogy and the principles of self-organization. *Behav. Sci.* 8:72. 10.3390/bs8080072 30096864 PMC6115738

[B76] Schlaug G. Altenmüller E. Thaut M. (2010). Music listening and music making in the treatment of neurological disorders and impairments. *Music Percept.* 27 249–250. 10.1525/MP.2010.27.4.249

[B77] Schlaug G. Norton A. Overy K. Winner E. (2005). Effects of music training on the child’s brain and cognitive development. *Ann. N. Y. Acad. Sci.* 1060 219–230. 10.1196/annals.1360.015 16597769

[B78] Schmithorst V. J. Holland S. K. (2004). The effect of musical training on the neural correlates of math processing: a functional magnetic resonance imaging study in humans. *J. Magn. Neurosci. Lett.* 354 193–196. 10.1016/j.neulet.2003.10.037 14700729

[B79] Schneider M. Siegler R. S. (2010). Representations of the magnitudes of fractions. *J. Exp. Psychol.* 36 1227–1238. 10.1037/a0018170 20873937

[B80] Seither-Preisler A. Parncutt R. Schneider P. (2014). Size and synchronization of auditory cortex promotes musical, literacy, and attentional skills in children. *J. Neurosci.* 34 10937–10949. 10.1523/JNEUROSCI.5315-13.2014 25122894 PMC6705250

[B81] Serrallach B. Groß C. Bernhofs V. Engelmann D. Benner J. Gündert N. (2016). Neural biomarkers for dyslexia, ADHD, and ADD in the auditory cortex of children. *Front. Neurosci.* 10:324. 10.3389/fnins.2016.00324 27471442 PMC4945653

[B82] Shadish W. R. Cook T. D. Campbell D. T. (2002). *Experimental and Quasi-Experimental Design for Generalized Causal Inference.* Boston: Houghton Mifflin.

[B83] Shalev R. S. Manor O. Auerbach J. Gross-Tsur V. (1998). Persistence of developmental dyscalculia: what counts? Results from a three-year prospective follow-up study. *J. Pediatr.* 133 358–362. 10.1016/S0022-3476(98)70269-0 9738716

[B84] Shalev R. S. Manor O. Gross-Tsur V. (2005). Developmental dyscalculia: a prospective six-year follow-up of a common learning disability. *Dev. Med. Child Neurol.* 47 121–125. 10.1017/S0012162205000216 15707235

[B85] Sigmundsson H. Anholt S. K. Talcott J. B. (2010). Are poor mathematics skills associated with visual deficits in temporal processing? *Neurosci. Lett.* 469 248–250. 10.1016/j.neulet.2009.12.005 19995594

[B86] Silva E. R. Baldin M. S. Santos F. H. (2017). Cognitive effects of numeracy musical training in Brazilian preschool children: a prospective pilot study. *Psychol. Neurosci.* 10 281–296. 10.1037/pne0000098

[B87] Skagerlund K. Träff U. (2016). Number processing and heterogeneity of developmental dyscalculia: subtypes with different cognitive profiles and deficits. *J. Learn. Disabil.* 49 36–50. 10.1177/0022219414522707 24598147

[B88] Spreen O. Strauss E. A. (1998). *Compendium of Neuropsychological Tests.* New York, NY: Oxford University Press.

[B89] Stein L. M. (1994). *TDE - Teste de Desempenho Escolar.* São Paulo: Casa do psicólogo.

[B90] Syah N. E. M. Hamzaid N. A. Murphy B. P. Lim E. (2015). Development of computer play pedagogy intervention for children with low conceptual understanding in basic mathematics operation using the dyscalculia feature approach. *Interact. Learn. Environ.* 24 1477–1496. 10.1080/10494820.2015.1023205

[B91] Thompson E. (2007). *Mind in Life: Biology, Phenomenology, and the Sciences of Mind.* Cambridge, MA: Harvard University Press.

[B92] Thompson W. F. Schellenberg E. G. Husain G. (2004). Decoding prosody in speech: do music lessons help? *Emotion* 4 46–64. 10.1037/1528-3542.4.1.46 15053726

[B93] van der Schyff D. Krueger J. (2019). “Musical empathy, from simulation to 4E interaction,” in *Music, Sound, and Mind*, ed. Corrêa A. F. (Rio de Janeiro: Editora da ABCM).

[B94] Varela F. J. Thompson E. Rosch E. (1991). *The Embodied Mind: Cognitive Science and Human Experience.* Cambridge, MA: The MIT Press.

[B95] Vaughn K. (2000). Music and mathematics: modest support for the oft-claimed relationship. *J. Aesthetic Educ.* 34 149–166.

[B96] Von Aster M. Dellatolas G. (2006). *ZAREKI-R - Batterie Pour l’évaluation du Traitement dês Nombres et du Calcul Chez l’enfant.* Paris: ECPA.

[B97] von Aster M. Shalev R. S. (2007). Number development and developmental dyscalculia. *Dev. Med. Child Neurol.* 49 868–873. 10.1111/j.1469-8749.2007.00868.x 17979867

[B98] Von Aster M. G. Schweiter M. Weinhold-Zulauf M. (2007). Rechenstörungen bei kindern: vorläufer, prävalenz und psychische symptome. *Zeitschrift für Entwicklungspsychologie und Pädagogische Psychologie* 39 85–96. 10.1026/0049-8637.39.2.85

[B99] Wan C. Y. Schlaug G. (2010). Music making as a tool for promoting brain plasticity across the life span. *Neuroscientist* 16 566–577. 10.1177/1073858410377805 20889966 PMC2996135

[B100] Wilson A. J. Dehaene S. Pinel P. Revkin S. K. Cohen L. Cohen D. (2006). Principles underlying the design of “The Number Race”, an adaptive computer game for remediation of dyscalculia. *Behav. Brain Funct.* 2 19. 10.1186/1744-9081-2-19 16734905 PMC1550244

[B101] World Health Organization [WHO], (2018). *The ICD-11 Classification of Mental and Behavioural Disorders: Diagnostic Criteria for Research.* Geneva: World Health Organization.

[B102] Zatorre R. J. Chen J. L. Penhune V. B. (2007). When the brain plays music: auditory– motor interactions in music perception and production. *Nat. Rev. Neurosci.* 8 547–558. 10.1038/nrn2152 17585307

